# Supplementary figures and images for: Spatiotemporal dynamics of signal dependent exocytosis and parasitophorous vacuolar membrane rupture during Plasmodium falciparum egress
Source: PLoS Pathog. 2026 May 11;22(5):e1014214. doi: 10.1371/journal.ppat.1014214 (PMC13183284; doi:10.1371/journal.ppat.1014214)

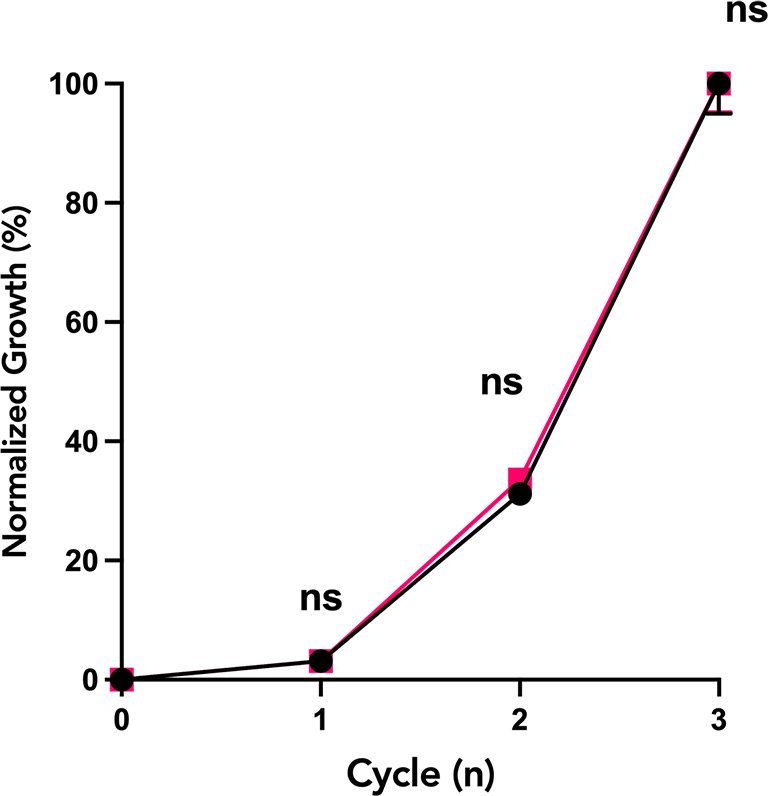

Supplement: S1 Fig — Parasitemia of PfEXP2mRuby3 (black) and PfEXP2mRuby3/PMXSEP (magenta) is measured via flow cytometry. Representative of 3 biological replicates. Each data point represents the mean of three technical repeats. (error bars = SD; not significant by unpaired t-test). (JPG) [file ppat.1014214.s001.jpg]

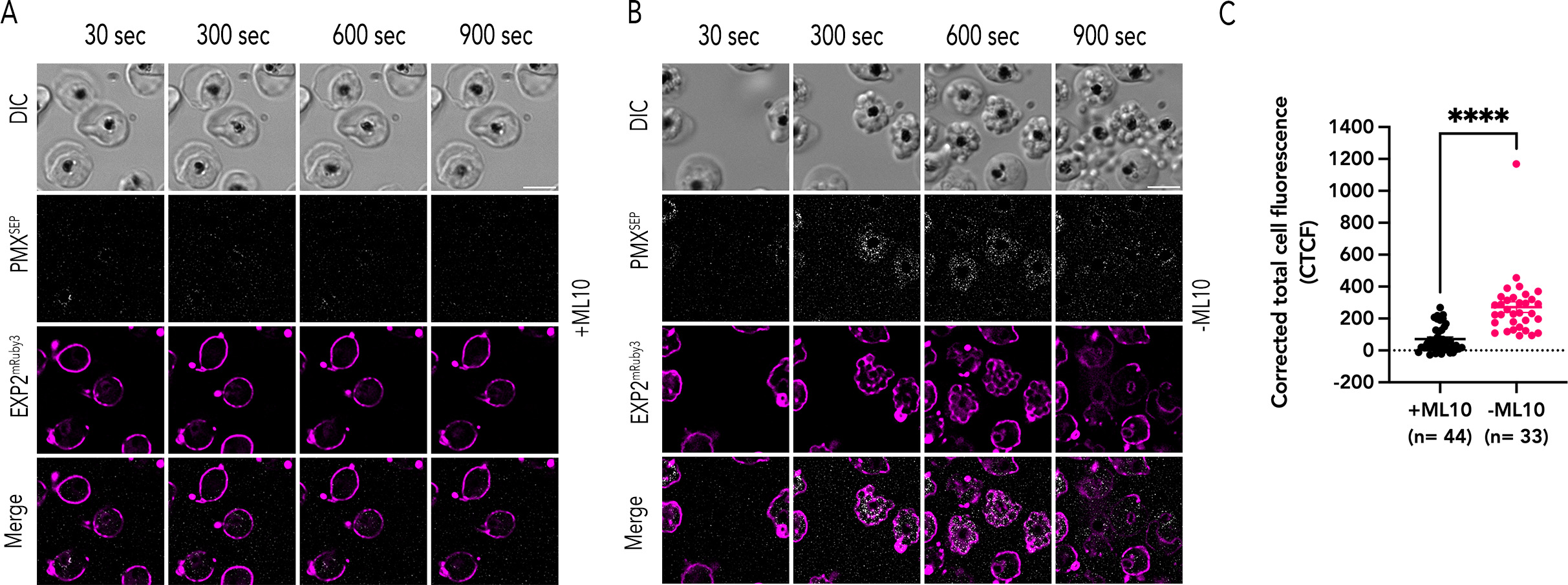

Supplement: S2 Fig — (A) A representative image from live imaging of synchronized PfEXP2mRuby3/PMXSEP schizonts in the presence of ML10 (B) A representative image from live imaging of synchronized PfEXP2mRuby3/PMXSEP schizonts after ML10 removal. Parasite egress occurs, and free-merozoites are scattered in the extracellular space (DIC). (C) Corrected total cell fluorescence (CTCF) of PMXSEP quantified from time-lapse images of synchronized PfEXP2mRuby3/PMXSEP schizonts incubated with ML 10 (black; n = 44, 2 biological replicates) or without ML10 (magenta; n = 33, 2 biological replicates) C1. ***p-value<0.0001, unpaired t-test. error bars = SEM Scale bar = 5 μm. (JPG) [file ppat.1014214.s002.jpg]

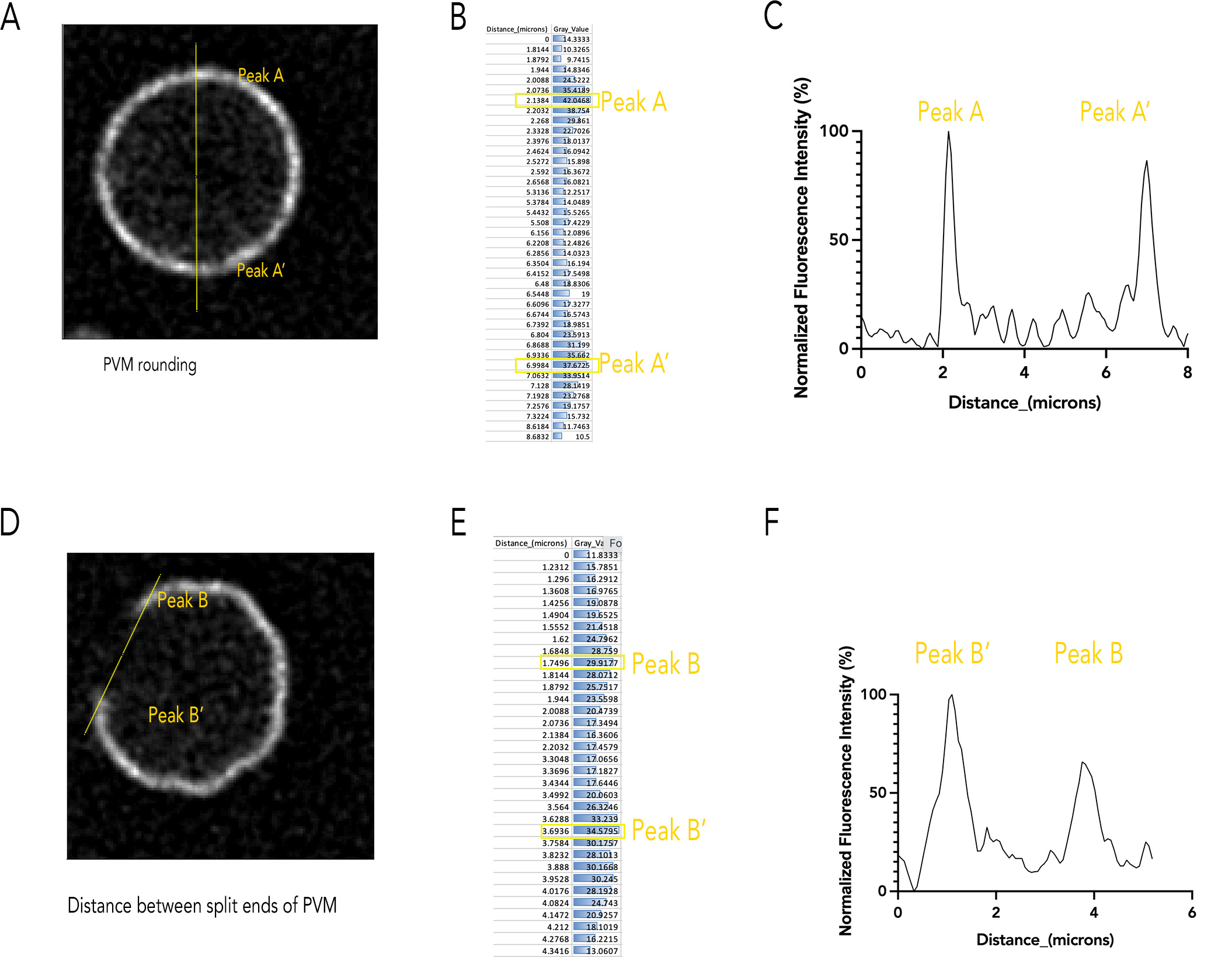

Supplement: S3 Fig — A representative of measuring PVM diameter (A-C). By drawing a line across the rounded PVM (A), measuring fluorescence intensity values corresponding to the lines are shown in (B), and generating a normalized fluorescence intensity graph in (C), the fluorescence intensity peaks are labelled as A and A’. A representative of measuring the distance between two split ends of PVM (D-F). Drawing a line across two split ends labeled as Peak B and B’ (C) and measuring fluorescence intensity values corresponding to the line (D) and its normalized fluorescence intensity graph generated (F). (JPG) [file ppat.1014214.s003.jpg]

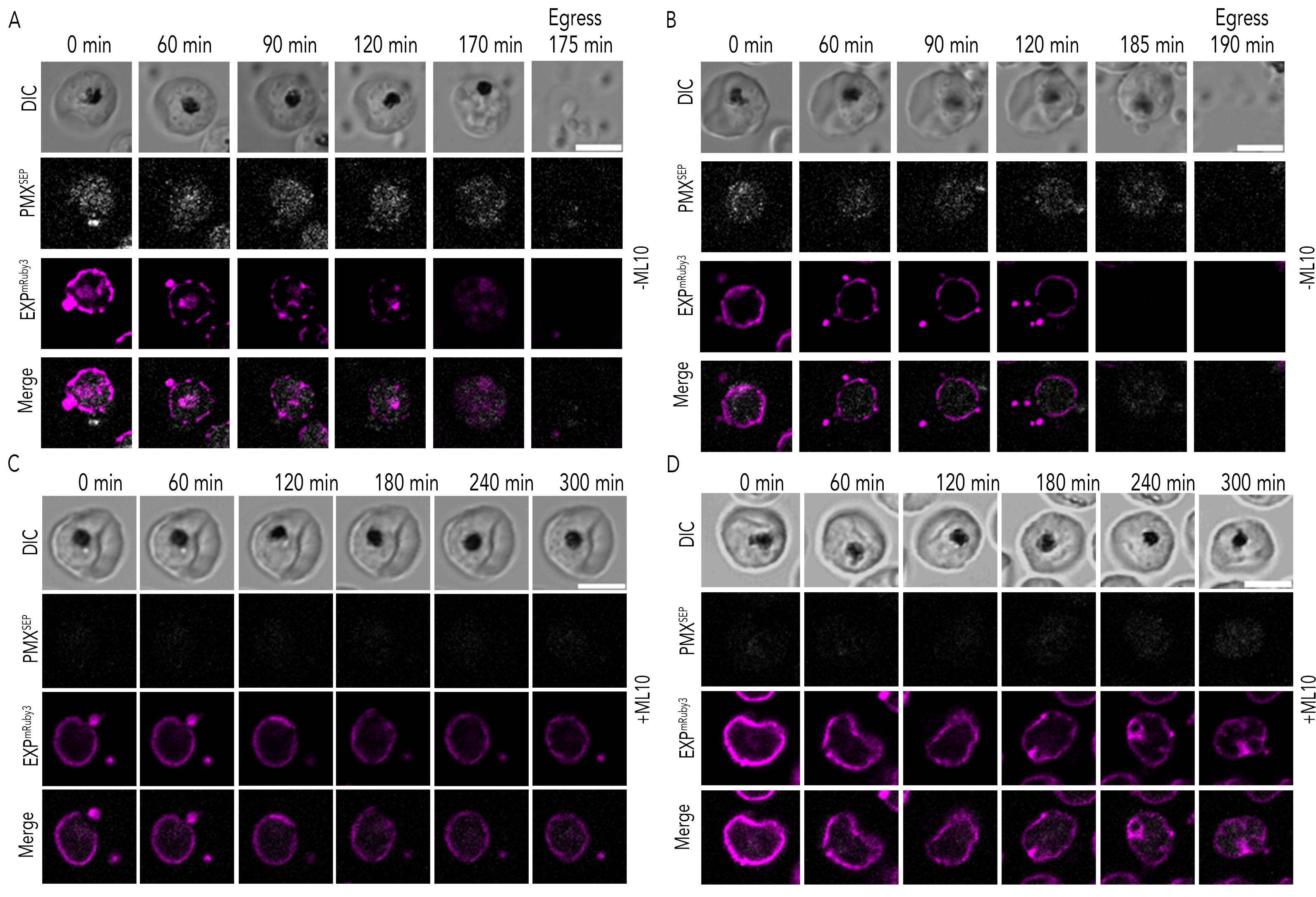

Supplement: S4 Fig — (A-B) Constant PMXSEP were detected at the 0 min till PfEXP2mRuby3/PMXSEP schizonts egress. Time-lapse images were taken at 5-min intervals. Scale bar = 5 μm; n = 1 biological replicates. (C-D) Representative images of live cell imaging of PfEXP2mRuby3/PMXSEP schizonts in the presence of ML10. All schizonts show no fluorescence detected throughout the 5-hour recording. Time-lapse images were taken at 5-min intervals. Scale bar = 5 μm; n = 2 biological replicates. (JPG) [file ppat.1014214.s004.jpg]

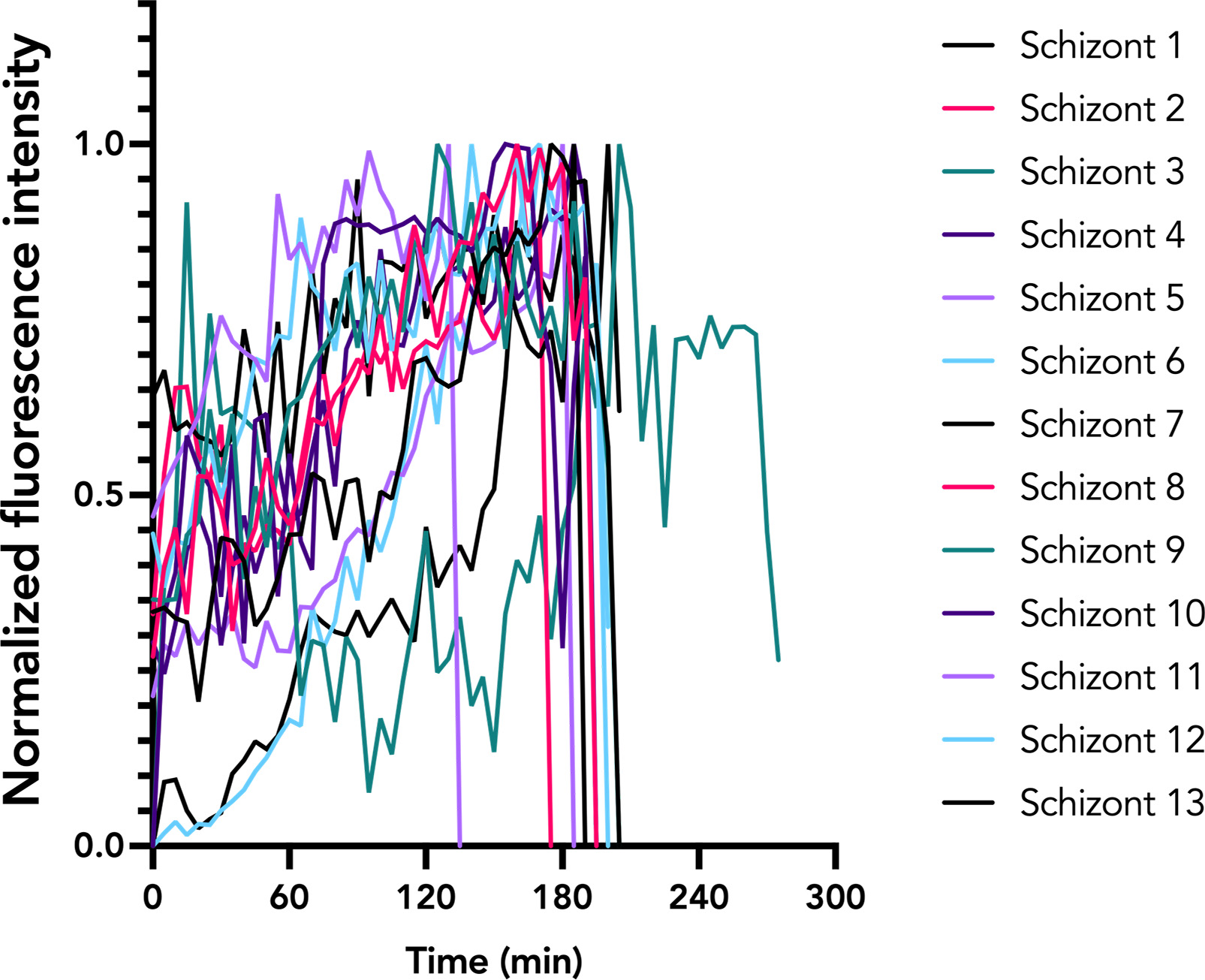

Supplement: S5 Fig — Corrected total cell fluorescence (CTCF) values were used to normalize against the highest CTCF of each individual schizont. Colors represent each individual schizont. (JPG) [file ppat.1014214.s005.jpg]

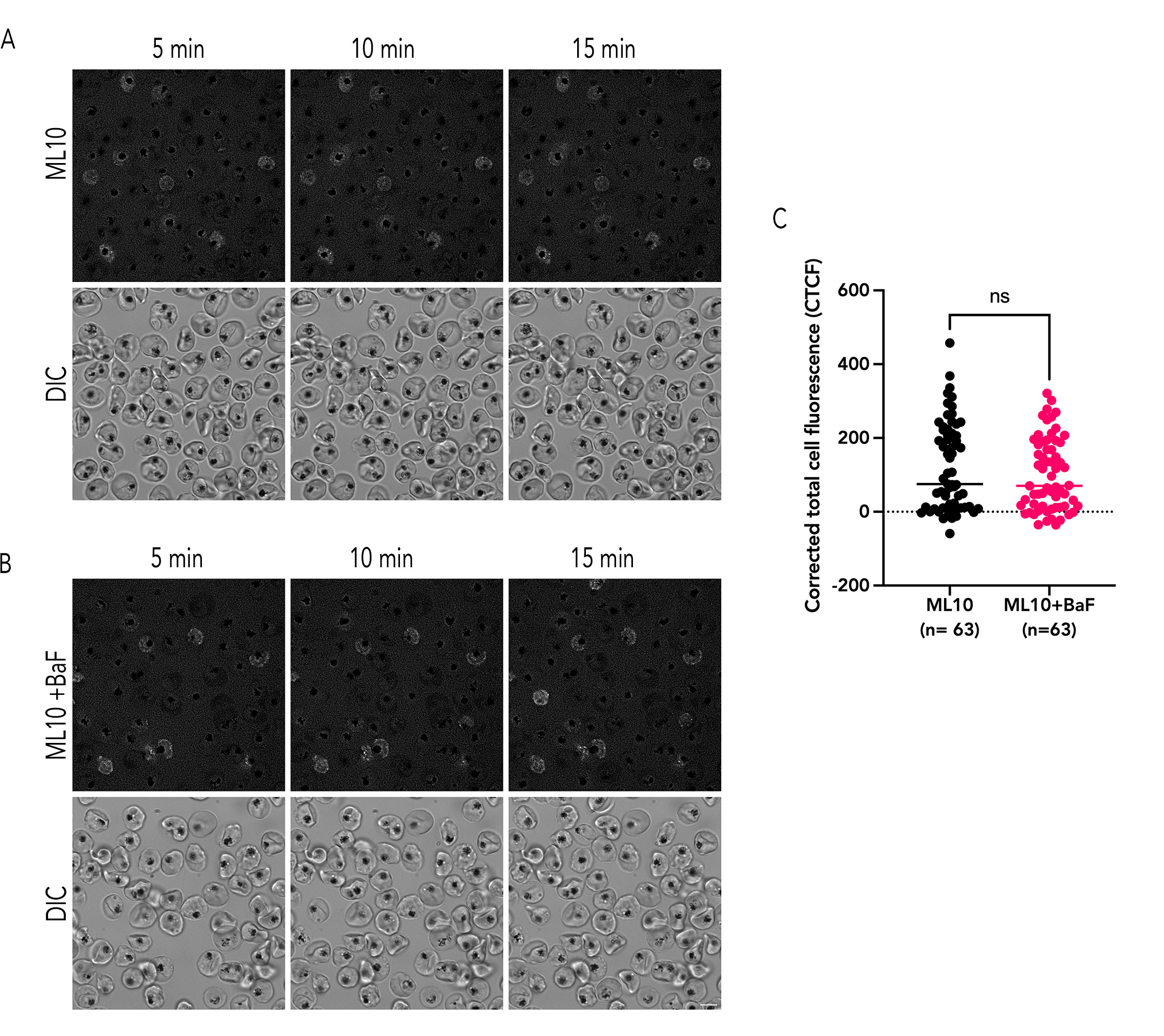

Supplement: S6 Fig — (A) A representative still image from a time-lapse video of ML10-treated PfEXP2mRuby3/PMXSEP late schizont and (B) A representative still image from a time-lapse video of BaF-ML10-treated PfEXP2mRuby3/PMXSEP late schizont. (C) Corrected total cell fluorescence (CTCF) values of PMXSEP schizonts. Black dot represents the CTCF value of each schizont with ML10 (n = 63, 3 replicates). Pink dot represents the CTCF value of each schizont in the presence of ML10 and BaF(n = 63, 3 replicates), ns = not significant, unpaired t-test Scale bar = 5 μm. (JPG) [file ppat.1014214.s006.jpg]

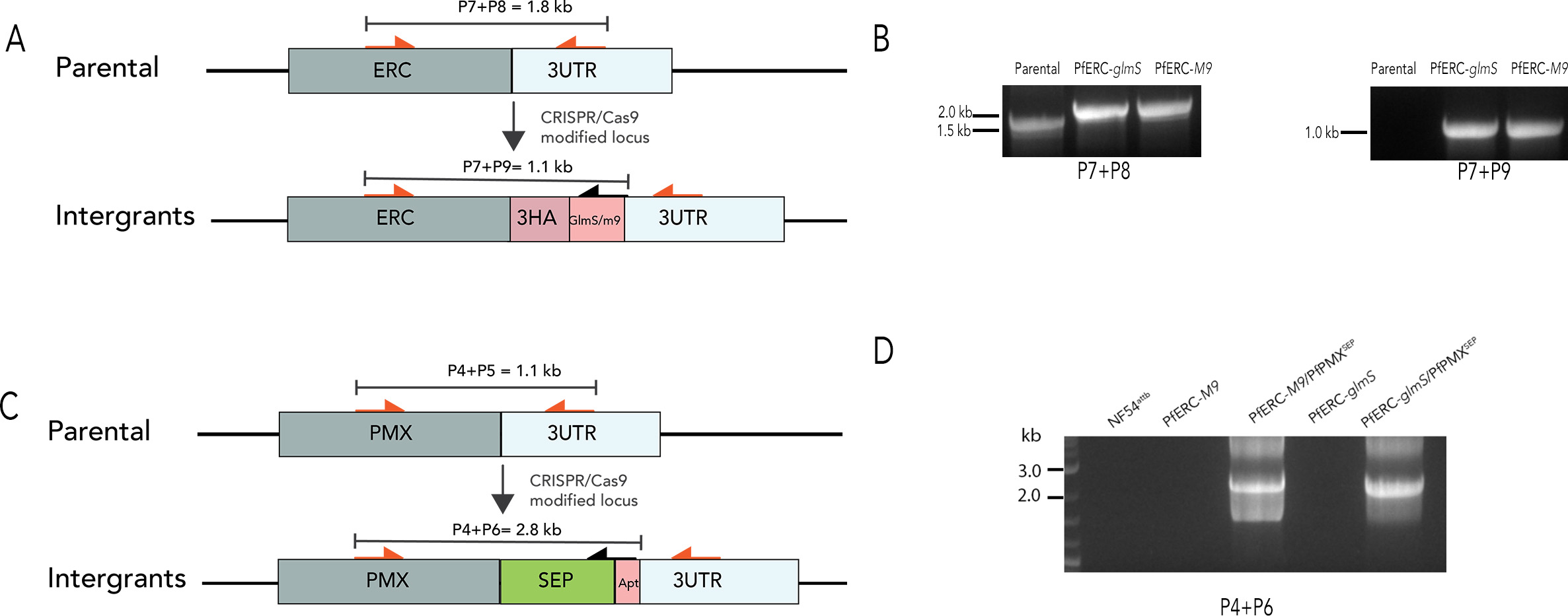

Supplement: S7 Fig — (A) Schematics of the targeting (pERC-TOPO) plasmids by the CRISPR/Cas9 system and a guide RNA. The locations of diagnostic primers (P7, P9, and P9) used to demonstrate the repair of the locus via double-crossover homologous integration are shown. (B) Agarose gel showing the PCR diagnostic test using the primer pairs P7 + P8 (left) and P7 + P9 (right). Amplicons were amplified from the genomic DNA extracted from parental and mutant parasites. (C) Schematics of the targeting pmx with SEP. (D) PCR diagnosis of SEP into the PfPMX locus in NF54attb, PfERC-M9, PfERC-M9/PMXSEP, PfERC-glmS and PfERC-glmS/PMXSEP using primer pair (P4 and P6). (JPG) [file ppat.1014214.s007.jpg]
