# Supplementary material for: Spatiotemporal dynamics of signal dependent exocytosis and parasitophorous vacuolar membrane rupture during Plasmodium falciparum egress
Source: PLoS Pathog. 2026 May 11;22(5):e1014214. doi: 10.1371/journal.ppat.1014214 (PMC13183284; doi:10.1371/journal.ppat.1014214)
Supplement: S1 Table — (DOCX) [file ppat.1014214.s008.docx]

| **Table S1. List of primers used in the study to generate the parasite lines** | | | |
| --- | --- | --- | --- |
| **Parasite line** | **Amplicon** | **Primers** | **Sequence 5’-3’** |
| PfEXP2^mRuby3^ | EXP2 Genomic F | P1 | TGCCTTAACCACCGTTATTAAAGATCC |
|  | EXP2-3UTR-AflII-R | P2 | TATTTCTCTCAAGGcttaagTCATAAGGAGAGTACATAAATAAAATAATC |
|  | mRuby3-R | P3 | GCATGGACGAGCTGTACAAGTGAcggccgcgtcgagtta |
| PfEXP2^mRuby3^/PMX^SEP^ | PMX-INT-F | P4 | GTAGGCTTAGGATTTCCAGGAATGTTATCAGCTGGGAATATACC |
|  | PMX 3' UTR R | P5 | GATGTAATAGGTTATACACTAGTATAATAAGTGTAACAAGCgatatcCCTTATGATGGG |
|  | Aptamer R | P6 | cctagtctagtttatataatatatttatgtactcacaatggggtctac |
|  | SEP-PsPXI-F | P10 | caaaaagtaaaaacctcgagggtatgagtaaaggagaagaacttttcactggagttgtc |
|  | SEP-Aat2-R | P11 | ggattacacatggcatggatgaactatacaaataagacgtctatccttatgacgt |
|  | U6 PMX guide F | P12 | taagtatataatattATTAATAATGCATTAAAAAAgttttagagctagaa |
|  | U6 PMX guide R | P13 | ttctagctctaaaacTTTTTTAATGCATTATTAATaatattatatactta |
| PfERC-*glmS*/PMX^SEP^ and PfERC-*M9*/PMX^SEP^ parasites | 3' ERC UTR R | P8 | gtatgtatatttgtaccccctattatctatccaatttgtcGCTAGCGAGCTCAAGG |
|  | 5' ERC C-Term F | P14 | AATTCGCCCTTTCCGCGGagaatagaaaaattatttcatttgatagataaaaacaatg |
|  | 3' ERC C-Term R | P15 | gacgatgcatcGcaacaaaaatccccagcaaTTGATGAATTAAGCGCTACTAGTTACCCA |
|  | 5' ERC UTR F | P16 | ATGATCTTGCCGGCAAGCTTtttatataaacatattttttttttttttaacataaaggg |
|  | 70xglmS-dia-pcr-R | P7 | gcaagatcatgtgatttctctttgttcaaggagtcaccccc |
|  | ERCgenomicF | P8 | GCTTCATATGTGTAATATTTTTGTTACATAAAAATGTGGTAAGATCAGGTGATAACATG |
|  | MKU6 ERC guide F | P17 | taagtatataatattCAATTGCTGGGGATTTTTGTgttttagagctagaa |
|  | MKU6 ERC guide R | P18 | ttctagctctaaaacACAAAAATCCCCAGCAATTGaatattatatactta |
