# Supplementary material for: Spatiotemporal dynamics of signal dependent exocytosis and parasitophorous vacuolar membrane rupture during Plasmodium falciparum egress
Source: PLoS Pathog. 2026 May 11;22(5):e1014214. doi: 10.1371/journal.ppat.1014214 (PMC13183284; doi:10.1371/journal.ppat.1014214)
Supplement: Attachment — Submitted filename: PeerReviewRespone.pdf [file ppat.1014214.s021.pdf]

Part I – Summary

Reviewer #1: Dedkhad et al. study P. falciparum exoneme exocytosis and PVM rupture, events that precede merozoite egress and invasion of new erythrocytes. The studies involve sophisticated imaging of several edited parasite lines, conditional knockdown of a key regulator enzyme, and relevant pharmacological reagents (C1, ML10, and BaF). The authors' transfectant lines are well-designed and have the potential to provide important insights into timing of key events (esp. discharge of exonemes, PVM ultrastructural changes and rupture, subsequent egress). Although the paper has excellent potential to advance our understanding of this critical, druggable stage in the malaria parasite life cycle, a number of issues require experimental controls and more thorough examination.

Author response: We thank the reviewer for their positive response to our submission and for their thoughtful feedback. In the resubmission, we have included more controls, specifically showing that neutralization of exoneme pH increases PMX^{SEP} fluorescence.

Reviewer #2: Dedkhad et al, describe the spatiotemporal dynamics of exoneme exocytosis and rupture of the PVM in relation to P. falciparum egress. The authors utilise a reporter (SEP) to visualise exoneme exocytosis in live P. falciparum schizonts by fusing it to Plasmepsin X, in a line where PVM breakdown can be in parallel monitored as EXP2, a known PVM marker, is fused to mRuby3. By using these tools, the authors confirm that exoneme exocytocis is PKG-dependent, as has been previously shown by other groups, and that PVM rupture occurs probably in a single site followed by full schizont egress within an average of 7 minutes. By using the SEP reporter the authors conclude that exoneme exocytosis occurs several hours prior to merozoite egress. Furthermore by using conditional genetics, they show that an ER-resident calcium binding protein (PfERC), which has been previously shown to be important for egress, is involved in exoneme exocytosis but not in the biogenesis of these organelles. Overall, this study provides new insight on the complex proteolytic and signalling cascade that leads to schizont egress in Plasmodium, however certain conclusions seem to be premature and key aspects need to be addressed before it is suitable for publication.

Author response: We appreciate the thoughtful and constructive feedback. The manuscript has been revised to include new data as well as controls that bolster some of our conclusions and toned down some of our conclusions that are not fully supported by the data.

Reviewer #3: The authors report a dual-reporter system to study the exocytosis of exonemes into the parasitophorous vacuole (PV) immediately prior to _Plasmodium_ egress. Egress is a rapid, multistep process that results in the rupture of the two bounding membranes: the PV membrane (PVM) and the red blood cell membrane (RBCM). Exonemal secretion is essential for this process,

as it delivers key effectors, most importantly the proteases PMX and SUB1, to the PV where they initiate a proteolytic cascade leading to egress.

While previous studies have assessed these events and the localisation of the molecules involved in them primarily by IFAs and electron microscopy, this reporter system enables live imaging of exocytosis. The authors achieve this by tagging PMX with the pH-sensitive fluorophore SEP, which fluoresces only upon exposure to neutral pH following exocytosis, and the PVM-bound protein EXP2 with mRuby3 to continuously visualise the PVM. This approach provides, for the first time, a live view of exocytosis and PVM dynamics just prior to egress.

Using this system, they show that exocytosis can begin as early as three hours prior to egress, confirm characteristic PVM dynamics like rounding consistent with prior studies, and suggest that "the PVM ruptures at a single site." The authors convincingly validate their system by demonstrating the absence of SEP signal when egress is stalled by PKG inhibition (which prevents initiation of calcium signalling) or in an ERC knockdown line (ERC being required for egress-specific vesicle formation).

Overall, I found the majority of the experiments to be robust, well-presented, and clearly described. The paper is well-written and easy to follow, and I recognise the considerable effort involved in live imaging such a challenging process. However, in some instances, the authors' interpretation seems to extend beyond the evidence presented, and additional controls or experiments would strengthen the conclusions.
Author response: We agree, we have toned our conclusions so that it is better supported by our data. More controls are also included in the resubmission to bolster some of our conclusions. We thank the reviewer for their overall positive assessment of our work as well as for their constructive feedback that helped make our study more robust.

Below, we have addressed all essential revisions and provided point-by-point responses to the reviewers' comments.

Reviewer #1:

1. The authors' proposal that exoneme exocytosis may be triggered as early as 3 h prior to merozoite egress contrasts with the prevailing view (model proposed in PMID: 28683142), where exoneme discharge leads to SERA5 processing and egress within 8 min. I understand that the authors used live-cell imaging that might capture exocytic events missed by prior studies.

However, a few considerations suggest more cautious interpretation is warranted.

a. The schematic in Fig. 1A shows the PMXSEP reporter initially producing a fluorescent signal upon packaging into nascent non-acidified exonemes; subsequent acidification causes loss of fluorescence. Fusion with the PPM neutralizes the fused vesicle to uncover SEP fluorescence as a transient punctum that dissipates due to dilution in the vacuolar space. As the kinetics of exoneme formation and acidification are unknown, it seems likely that PMXSEP packaging and subsequent exoneme acidification will produce a similar transient punctum. Moreover, because PMX transcription peaks around 40-42 h (per DeRisi lab microarray data on PlasmoDB), this reviewer expects to see fluorescence puncta associated with packaging.

Author response: We agree with the reviewer that the live-imaging experiment monitoring PMX^{SEP} packing and followed by exoneme vesicle acidification, will produce a punctate signal. Since the kinetics of exoneme biogenesis and acidification are lacking, we used the microarray data available on PlasmoDB to set the observation time frame that coincides with PMX mRNA expression. However, PMX transcription peaks vary across the studied Plasmodium strains. In fact, no NF54 data set is currently available and as the reviewer is surely aware, mRNA expression and protein expression need not coincide (and often do not in the case of *P. falciparum*). Another challenge comes from the sensitivity of the microscopes. We made several attempts to capture vesicle acidification using time-lapse live imaging around the time when PMX transcription peaks, but we have not succeeded in capturing these events reproducibly. We only captured only one schizont undergoing vesicle acidification (around 40 h.p.i). We hypothesized that exonemes have already acidified when we perform live imaging on 44 h.p.i schizonts. To determine if PMX-containing vesicles are already acidified at the time of our observations, we decided to use a weak-base ammonium chloride to neutralize the pH of acidic exonemes (Fig. 5D, E). Ammonium chloride is used routinely to raise the pH of intracellular compartments [1]. These data show that PMX^{SEP} signals are increased upon the addition of ammonium chloride at the start of our observations at 44 hpi. These data suggest that the fluorescence events we observe are not PMX^{SEP} packaging and exoneme acidification since addition of the weak base would have no effect on PMX^{SEP} fluorescence if it was present in immature non-acidic vesicles. These new data are shown in Fig. 2E, F and Fig. 5D, E.

b. Comparison of Fig. 2A and 2D shows that Compound C1 completely blocks invasion (Fig. 2A) but only partially reduces cellular fluorescence (Fig. 2D). Given the critical role of PKG in exoneme discharge, this is very surprising. To this reviewer, ongoing fluorescence speckles in the presence of C1 appears to reflect synthesis and packaging of PMXsep into new exonemes and subsequent loss of fluorescence due to organelle acidification. Can the authors devise experiments that exclude this possibility?

Author response: As seen in Fig. 2D, there are a few outliers that showed some PMXsep fluorescence in the presence of C1. However, quantification of the fluorescence demonstrates that this is a minor subset (Fig. 2D). It is also possible that these fluorescence speckles are from schizonts that died (and thus lost their ability to acidify compartments) upon incubation with C1. Another possibility is that these outliers showing PMXsep fluorescence had incomplete inhibition of PKG either due to higher expression of PKG or these schizonts were deficient in taking up the PKG inhibitors into cells. Newly included data show that incubating these age-matched schizonts (48h.p.i) with a weak base like ammonium chloride results in a similar increase in PMXsep fluorescence strongly suggesting that the exonemes present at this lifecycle stage are fully acidified (Fig. 2E,F). Together, these show that the secretory vesicles were acidic at the time of our observations (44 and 48 h.p.i) as seen in Fig. 2E, F and in Fig. 5D, E.

2. Comparison of EXP2mRuby3 signals in Fig. 2B vs 2C shows a marked and unexpected increase in red fluorescence when C1 is removed. Comparison of panels A and B in Supplementary Fig. 1 reveals a similarly marked increase upon ML10 removal. Do the authors have an explanation for this?
Author response: The differences in brightness of EXP2mRuby3 signals came from the brightness and contrast adjustment. The signals were not included in any fluorescence intensity measurements. We have adjusted brightness and contrast equally to avoid confusion for the reader. These data are presented in Figures 2B and 2C.

The Methods states "The adjustments to brightness and contrast were made only for display purposes.", but I assume this does not account for the change in red fluorescence.
Author response: The reviewer is correct. We adjusted brightness and contrast across all fluorescence and brightfield channels for display purposes. These adjustments actually do account for the change in red fluorescence and were corrected in the resubmission.

3. Along the same line, could the authors clarify how CTCF is calculated?
Author response: Yes. We have included more detail in the methods section to help clarify the process (also included here below).

"The fluorescence intensity of each cell was calculated using the calculation for corrected total cell fluorescence (CTCF) = integrated density–(area of selected cell × mean fluorescence of background readings) [2,3]. The data for calculation were obtained only from the SEP fluorescence channel as follows*: 1) From the Analyze menu on ImageJ, set measurements were selected with the area, min, max gray values, and mean gray values. 2) The area of the cell of interest was manually selected using a freehand tool. 3) The selected area was measured using*

Measure. 4) Step 2-3 was repeated for every time point. 5) For background readings, an outline was drawn on the space outside the cell of interest. 6) The measure menu was used to measure fluorescence as described above. 7) The fluorescence intensity of each cell was calculated using Excel (Microsoft® Excel for Mac Version 16.103 (25110922)). Plots and statistical analysis (2-sided unpaired Student t-tests) were done using GraphPad Prism (Prism 10 for macOS Version 10.2.2 (341). "

The Methods section does not explicitly state that the total cell fluorescence is bandwidth-limited to quantify SEP fluorescence and does not include mRuby3 fluorescence. If it includes both, the data in Fig. 2D and Supp. Fig. 1C will need to be reanalyzed.
Author response: We apologize for the confusion. The mRuby3 fluorescence was not included in the calculation of the SEP fluorescence signal. We have revised the text to state this explicitly as well as include more detail on how the fluorescence was quantified (see methods and above).

4. Lines 157-162. ML10 and C1 are not unrelated as this text implies. Both contain the diaryl-(4-piperidinyl)-pyrrole scaffold. ML10 was developed from a med-chem project based on C2, which was developed from chemistry around C1. Both compounds have very similar binding sites on PKG based on mutations in a small gatekeeper residue (PMID: 33391223). There is value in testing both compounds, but they *should not be* considered independent tests of the authors' model.
Author response: We thank the reviewer for catching this mis-statement. The intention in using C1 and ML10 to perform a similar experiment was to show that there was no difference between the PKG inhibitors. We have modified the statement in our results to reflect this more accurately.

5. Fig. 4. Line 227 should be more cautiously worded; also at lines 441-443. Rather than being a regulated process with a specific site, it is possible that PVM rupture at one site dilutes the lytic factors, halting further digestion of the PVM at its inner face and preventing formation of a second rupture pore.
Author response: We have modified the conclusions to include this caveat.

6. Supp. Fig. 3 and Movie S7. Complete loss of PMXSEP fluorescence development in imaging of cells for over 5 h upon ML10 addition contrasts with the results with C1 in Fig. 2D, where fluorescence development continued in a subset of cells blocked with C1. As stated above, PMX transcription peaks around 40-42 h (per DeRisi lab microarray data on PlasmoDB), leading me to expect fluorescence speckles when new PMX is synthesized and packaged into exonemes despite complete PKG block by ML10. To this reviewer, these data suggest that ML10 may have an off-target effect. For example, it may interfere with PMX translation and packaging into new

exonemes or it might block SEP fluorescence development. Although touted to be specific, PKG mutants where the binding pocket is blocked are still killed with a growth EC50 of ~ 100 nM (see Fic. 1C of PMID: 32673324), revealing that there is some off-target toxicity associated with this compound. Can the authors perform control expts to exclude ML10 effects on PMX translation/packaging and on SEP fluorescence development?
Author response: While it cannot be ruled out that ML10 may have an off-target effect, ML10 concentration at 25 nM was shown to be highly specific to PKG [4–6]. Newly included data using the ammonium chloride shows that we observe an increase in PMXsep fluorescence in the presence of ML10 showing that ML10 has no effect on SEP fluorescence.

7. The inability of bafiloymycin A1 (BaF) to increase PMXSEP fluorescence is unexpected and interpreted by the authors to implicate exoneme acidification by a mechanism other than the V type H+ ATPase pump. As this experiment was only performed in the presence of ML10, I consider this to support the above off-target effect of ML10 on SEP fluorescence development. Without excluding such an effect, the authors may want to avoid statements about mechanisms of exoneme acidification (esp. lines 389-399 but also lines 283-284).
Author response: We agree that the inability of BaF to change exoneme pH was a surprising observation, one we did not expect either. Our data in Fig. 2 and Fig. 5 shows that ML10 has no effect on SEP fluorescence which is observed when we neutralize the pH in the secretory vesicles using ammonium chloride. These data bolster our surprising finding that BaF has no effect on acidification of exonemes.

8. Fig. 6B. These immunoblots compare ERC and PMX band intensities between PfERC-glmS/PMXSEP and PfERC-M9/PMXSEP after application of 7.5 mM GlcN for 48 h. While the M9 mutant ribozyme is a good control for glmS-mediated knockdown, it does not permit quantification of knockdown in the glmS line. Matched cultures with and without glucosamine (GlcN) treatment for the PfERC-glmS/PMXSEP are required to assess extent of ERC knockdown and to evaluate PMX processing. Such matching with the M9 mutant will also allow assessment of GlcN toxicity, which has been observed in many labs that use lower GlcN concentrations (e.g. PMID: 39829323).
Author response: We disagree that parasites with and without GlcN are better controls especially because of the off-target effects of GlcN. It is standard in the field to compare M9 with glmS matched parasite lines. The M9 is a mutated glmS ribozyme wild type that serves as a more robust control when using GlcN treatment in several studies [7–9]. There is no report on GlcN toxicity on the NF54 parasite line but on the CS2 line that has been selected to express specific PfEMP1[10–12]. To rule out GlcN toxicity or off target effects on SEP fluorescence, we used M9

mutants as controls. Furthermore, the prior study on PfERC showed that PMX expression was identical between M9 and glmS parasite lines in the absence of GlcN [8].

Reviewer #2: My biggest concern is that the authors base all of their conclusions on live video microscopy without any biochemical data to support these claims and also that the protein selected for fusing SEP could lead to the wrong conclusions regarding the timing of events. In a recent paper by Triglia et al, (2023) it was shown that PMX has a dual localization in both exonemes and micronemes as it processes a series of proteins and proteases in both compartments. With this in mind, these results point to both exonemes and micronemes discharging their contents or have the pH changed due to an unknown mechanism or proton pump?
Author response: We agree that further biochemical data are required to clarify the model, though this is beyond the scope of this manuscript and will be addressed in future studies. The current data supports a clear role for PMX in parasite egress and therefore, we would argue that it is a valid target for SEP fusion. Furthermore, our attempts to fuse a live fluorescence reporter to SUB1 has not yet been successful. The work by Triglia et al showed that PMX is required for maturation of Rh5 and blocking exocytosis by C1 leads to an alternative cleavage of Rh5, which they interpret as PMX being in micronemes.
Similarly, Mukherjee at al (Nat. Commun. 2022) show PMX cleaved other invasion ligands and used both EM and super-resolution microscopy to understand how PMX gains access to invasion ligands within the merozoite. They show that PMX overlaps with invasion ligands as well as with SUB1. Similarly, SUB1 overlaps with invasion ligands as well. However, since both micronemes and exonemes are made from the same post-Golgi compartments, Mukherjee et al reached a more careful interpretation of their data that PMX likely encounters and cleaves invasion ligands in this precursor compartment. Given the size of merozoites and limitations of microscopy, it is difficult to distinguish between micronemes and exonemes and more careful studies are needed to determine how PMX gains access to invasion ligands prior to secretion. We have included these caveats in the revised manuscript though given the limitations of our live microscopy data, we would like to refrain from suggesting both compartments are refractory to BaF and therefore may have novel mechanisms for acidification.

SUB1 could have been a better target if genetic manipulation is possible and tagging does not interfere with function.
Author response: While we agree with the reviewer that SUB1 could be another target given its specific location within exonemes, we focused this paper on the unknown timeline of exoneme exocytosis. The current literature supports PMX as a suitable target because it is the most

upstream protein in the protease cascade. Our previous attempts to fuse a live fluorescence marker to SUB1 failed and hence we focused this study on PMX.

Are there any other exoneme markers that could be tagged to verify the data?
Author response: Plasmepsin X and SUB1 are the two best characterized exoneme proteins. Unfortunately, there are no other characterized exoneme markers.

Regarding the timing of exocytosis, the authors state that "Another possibility is that the observed PMXSEP fluorescence at this earlier timepoint could be secretory vesicles that were not yet acidified and therefore fluorescent." (lines 262-263). So why do the authors believe that their data point to the time of release and not lack of acidification?
Author response: Newly included data using ammonium chloride is inconsistent with this hypothesis. Therefore, we have removed this line from the manuscript.

How can the authors exclude that these might be micronemes (yet to be acidified) where PMX resides? regarding PMX might be located to micronemes,
Author response: The reviewer is correct. PMX is localised to multiple apical vesicles [13,14]. It has been a challenge in the field to physically differentiate the microneme and exoneme without known markers and high resolution microscope. This is the case for SUB1 [15] as well.

What is the pH of the PV? Has this ever been measured?
Author response: A direct pH measurement of the PV is lacking. The data suggest that the pH of the PV is neutral pH comes from a study of SUB1 activation. SUB1 is activated upon discharge into the PV at neutral pH [16].

Additional more specific points:
1. Lines 111-112 and Figure 1E: The fact that the bright puncta are inside the PVM does not mean that this is secreted PMX in the PV. One could argue that these reside inside the merozoite. Have the authors performed any IFAs and/or expansion microscopy to show localisation of PMX at this stage?
Author response: Our intent was to demonstrate that the two fluorescence tags were expressed and not mislocalized. We have toned this conclusion down to suggest that the likely localization upon PMXsep exocytosis is the PV. We carefully designed this parasite line to support our live imaging efforts and answer specific questions about exocytosis in schizonts as well as during egress. Thus, PMX was tagged with a pH-sensitive GFP. We tried several anti-GFP antibodies but failed to find a suitable antibody for PMXsep that allowed us to observe this fusion protein

via expansion microscopy or IFA. Furthermore, only a small fraction of PMX is secreted at this time and even with IFA, it is likely that the overwhelming signal from the unsecreted PMX within vesicles will mask any PMX secreted into the PV.

How late is that schizont and a more general comment, how do the authors distinguish between a 45h and a 47h schizont by live microscopy?
Author response: Characterizing a 45 h and a 47 h schizont by live microscopy is challenging and impossible since the cell division is an asynchronous process. We did not use live microscopy to distinguish the time of schizogony. Instead, we tightly synchronized parasites by using C1 or ML10 to prevent egress, then washed off the compound. After allowing parasite egress and invasion for 2 hours, we perform 5% sorbitol on the culture to collect 0-2 rings. Using this window, we count the hours post infection for all schizonts. These details are included in the methods.

Was this culture treated with C1 or ML10?
Author response: Parasites were treated with C1 or ML10 for 3-4 h for experiments that require tightly synchronized parasites. Upon C1 or ML 10 being washed off, parasites were grown in the suitable media without C1 or ML 10.

2. Lines 148-149 and Figure 2C, Movie S2: The authors state "This is followed by rapid dissolution of the fluorescence due to the rapid dilution of PMXSEP into the much larger PV compartment." This contradicts the previous statement they made and looking at the figure and the movie, the rapid dissolution occurs after RBC rupture. In Fig.2C (panel 600 sec) the bright schizont in the middle has clearly the PVM broken. This discrepancy between the text and the Figures is even more evident in Fig.3A where intensity seems to be increased in the -30 sec panel compared to the -390 sec where the authors show PVM rupture. So PMX is clearly visible throughout the whole process of PVM rupture. Can the authors clarify their findings here?
Author response: There are multiple vesicles with PMX being secreted, what we mean is that once a vesicle is secreted, the PMXsep fluorescence from within that vesicle is lost as it dilutes into the much larger compartment of the PV. We have further clarified this text.

Furthermore it would have beneficial for the reader if the authors included the SEP panel in Fig.4.
Author response: Figure 4 provided data on PVM rupture. The microscopy we used is limited to a 4-second interval with two channels; one fluorescence and one brightfield channel, or a 2-second interval with one fluorescence channel, to achieve good image quality. Thus, we couldn't capture the SEP fluorescence due to these limitations.

I appreciate the aim of this section is to study PVM rupture, however the hypothesis that it occurs at a single site using EXP2-mNeonGreen line was already been proposed and in part shown by Glushakova et al, 2019. Are there technical limitations in imaging SEP every 2 or 4 seconds? If yes, then this section should be supplementary data.

Author response: The reviewer is correct that the study by Glushakova et al (2018) proposed the hypothesis that PVM rupture occurs at a single site. The study conducted thorough live-cell imaging of PVM morphological changes and rupture, as well as merozoite egress, provided a timeline for the current model. However, neither Figure 3A nor movie S4 clearly showed a single PVM rupture site [17]. We believe our data provides key evidence supporting the previously proposed hypothesis that PVM occurs at a single site. Thus, we would prefer to keep these data as presented.

3. If the authors suggest that exoneme release occurs 3h prior to egress, that would mean that PMX and SUB1 are in the PV. Have the authors performed any Western blots with cytosolic fractions to show presence of either protease?

Author response: Currently, we don't have anti-PMX or anti-SUB1 available. We have tried several anti-GFP antibodies, including rabbit anti-GFP (Life Technologies: 11814460001), mouse anti-GFP (Roche Diagnostics: A-6455), rabbit anti-GFP (Life Technologies: G10362), and mouse anti-GFP (JL-8) (Clontech: 632381), to detect the fluorescence SEP protein. We found that none of them were sensitive enough to detect SEP expression. However, the mouse anti-GFP (JL-8) could detect the SEP but required a lot of material and was not sensitive. To detect SEP expression, ~10^8 cells/mL was required. Our data suggests that the amount of PMX in the PV at 3h prior to egress would be minimal and hence we would require a much more sensitive detection method rather than a western blot to detect the minimal amount of PMX in the PV at that time. Thus, we could not perform the Western blot at the early time point, when PMX is just beginning to be released into the PV.

Also this would mean that MSP1 and SERA5/6 would be processed albeit not fully if we assume that it is a slow process. A combination of IFAs and Western blots are needed to validate the above observations.

Author response: It may not be possible to detect these early processing events via western blot. The data presented in this manuscript lays the groundwork to pursue other avenues of studies on exactly how the parasite sets up its egress but these are beyond the scope of this present work. Even though PMX is secreted, enough protease may not be present at these early time points OR a second factor such as MSA180 [18] may be required for processing of SERA5/6 OR as the reviewer suggests it may be a slow process. Thus, these experiments would likely not be conclusive.

Reviewer #3: Validating the reporter system

Overall, the absence of PMX-SEP signal when PKG is inhibited and when ERC is knocked down suggests that what we see are authentic events of exocytosis of PMX-carrying exonemes. However, confirming the model of action in Fig 1A in the presented data is challenging as we are unable to visualise the vesicles fusing to the parasite's plasma membrane (as the authors themselves consider). Since this live reporter system has potential to be a great tool for studying egress, I think more controls are required for validating "tagging the pmx locus with SEP did not affect its biogenesis, maturation, function, or protein localization" and that " punctate PMX-SEP fluorescence within the PVM, indicating PMX_SEP was exposed to neutral pH and suggesting that exoneme exocytosis had occurred" (no data shown directly proves what we see are exonemes undergoing exocytosis). These could perhaps help? –

1) A PMX-GFP line that clearly shows PMX localisation in exonemes throughout irrespective of C1 treatment and whose localisation should not show the dynamics of a pH sensor.

Author response: We agree that tagging PMX with non-pH-sensitive fluorophores could offer greater value for monitoring subcellular localization. However, without co-expressing with a known exoneme marker in the same parasite line, PMX-GFP live-imaging would not resolve the subcellular localization. The other issue is that the only other validated exoneme marker, SUB1, has so far not proven to be amenable to fusion with a fluorescent protein. Our argument was that tagging with SEP did not interfere with the normal function of PMX because if that was the case, these parasites would not survive because the function of PMX is essential for the parasite. We have edited it for clarity.

2) IFAs to confirm exonemal and later on PV localisation of PMX-SEP. This will bridge the gap between PMX localisation data shown in previous studies (like Mukherjee et al., 2022) and this report.

Author response: The IFA to confirm exonemal and PV localization of PMX-SEP poses a technical challenge due to the lack of known and validated exoneme antibody. Further, given the relative amounts of PMX present in exonemes vs the PV, the concentrated signal from the vesicles would mask any signal from the PV. This is a similar issue faced by Mukherjee et al where they failed to observe PMX in the PV. Resolving the PV signal will require brighter fluorophores than what has been utilized previously.

Also another related suggestion - Did the authors try live imaging in the presence of E64 inhibitor that allows exonemal release but stalls the final step of RBCM rupture. This might allow us to visualise exocytosis and PVM rupture better as the merozoites will be contained within the RBC?

Author response: We are unclear what the reviewer means by “to visualise exocytosis and PVM rupture better”. It is correct that in the presence of E64, merozoites will be contained within the RBC after PVM rupture. However, it’s unlikely that using E64 would improve visualization of exoneme exocytosis and PVM rupture. Alternatively, high-resolution microscopy, such as spinning disk confocal microscopy or lattice light-sheet microscopy (LLSM) [19] would help address this issue. Future studies using these tools would help refine the model for egress.

Presentation of live imaging data

One limitation I noted is that many of the live imaging datasets are presented as single-schizont examples, without showing the full field of view. Whilst Fig 2, supplementary videos 1 and 2 and Fig 6, supplementary videos 8 and 9 show multiple schizonts in the field, all other data are restricted to just one schizont. Since the plotted graphs presumably come from observing several schizonts within larger fields of view, it would be helpful if representative full-field videos were made available as supplementary. This would allow readers to better assess the uniformity of the reported observations. This is particularly important in relation to the discussion below on PVM rupture.

Author response: We have added more videos as the reviewer suggested.

Also, minor point relating to this- for all time-lapses, would it be possible to plot normalised SEP fluorescence intensity vs time for each schizont until egress? Some videos suggest there might be some peak exocytosis activity a few secs before egress, so would be interesting to see if there is a trend there. This would also be a better representation of data shown in Fig 5B. Author response: We did plot normalized SEP fluorescence intensity but did not observe any patterns or specific peaks. Hence we did not include it in the original submission. Those data are included in the supplementary data now (suppl. Fig. 4). In some schizonts there are hints that exocytosis levels increase with time (5 out of 13), however, this was not the case for all and neither were specific peaks observed at specific times before egress.

PVM rupture at a single site.

The observation of PVM rupture at a single site in the presented videos is striking. Could the authors clarify whether this was uniformly observed across all schizonts undergoing egress within the full fields of view?

Author response: Yes, this was observed for all schizonts undergoing egress. All 74 schizonts observed over several biological replicates showed PVM rupture at a single site.

Only 13 schizonts are quantified — what was the outcome for the others? Author response: Some schizonts egressed but were not selected because they were out of focus during imaging. Several schizonts moved out of the field of view and did not adhere to the glass

bottom dish. Some schizonts did not egress during the observation time frame and hence were not utilized in the analysis.

If rupture does indeed occur at a single site, why is it consistently observed at the side of the parasite? Given that parasites should be randomly oriented, one might expect some events where rupture occurs above the focal plane, following widening of that rupture, which when it reaches the focal plane of view would appear as a more uniform disappearance of the PVM. Alternatively, could the apparent single-site rupture reflect physical constraints of the setting — for instance, parasites bound to poly-lysine–coated slides or surrounded by neighbouring schizonts, which might restrict exit to one side?
Author response: This is because we are using widefield microscopy and the focal plane makes the signal strongest around the circumference of the parasite. Hence, we do not believe that crowding or the poly-lysine would have an effect on the PVM rupture as there is an RBC cytoplasm protecting the PVM from these outside physical interactions. While the PVM ruptures at a single site, this site appears to be randomly oriented all around the circumference. At this time we do not have a landmark at the PVM to decipher if the rupture site is specific or random. Future work, perhaps by labeling the cytostome to provide a landmark at the PVM, may help resolve this question.

It is indeed puzzling that release does not appear to occur through multiple points, given that the known molecular players appear to dismantle components globally (SERA6 dismantling beta-spectrin, Thomas et al.,2018).
Author response: We agree and future studies will help determine exactly how this is achieved.

It may be helpful if the authors could clarify their use of terms 'rupture' and 'poration', as they appear to be used somewhat interchangeably. These could represent distinct steps. Prior work has suggested that PVM rounding may be associated with or a consequence of poration that precedes PVM rupture (Tan & Blackman, 2021). It is not entirely clear whether the observed break in EXP2-mRuby signal reflects a single opening (pore) or the focal plane view of a broader rupture event.
Author response: Our current microscopy resolution prevents us from distinguishing from a single pore vs the focal plane view of a broader rupture event. We have used rupture instead of poration now to clarify the limitation of the tools.

Part III – Minor Issues: Editorial and Data Presentation Modifications
Please use this section for editorial suggestions as well as relatively minor modifications of existing data that would enhance clarity.

Reviewer #1: 1. Abstract (line 24) and Introduction (lines 82-83) are vague about the second "fluorescent marker on the PVM". This reviewer was left confused and wondering. I suggest describing that this as c-terminal tagging of EXP2 with mRuby or a GFP derivative so that readers can better anticipate how PVM rupture was tracked.
Author response: Changed.

2. "However, the spatiotemporal regulation of exocytosis, PVM breakdown, and merozoite egress remains unknown." This statement is a bit unfair as there is much known about exoneme discharge, timing of SUB1-mediated proteolysis of targets, subsequent PVM breakdown and egress, all occurring within ~ 8 min. Even if the authors perform control experiments and properly establish that occasional discharge of a few exonemes begins as early as 3 h before merozoite egress, their data suggests (and I assume they would agree) that the majority of exonemes are discharged shortly before the 8 min cascade of events that culminate in merozoite egress. different experiment tho.
Author response: We have edited the statement. While we agree that the 8 min timeline has been established, this timeline has been determined via studies that utilize inhibitors. This timeline has not been investigated without inhibitors nor has exocytosis been directly observed via microscopy. Our experiments suggest that the discharge is constant over the 3 hour timepoint, which opens up new avenues for investigation to determine the spatiotemporal regulation of egress.

Isn't it better to assume that rare exoneme discharge events occurring up to 3 h before egress are spurious cellular events and that quantitative discharge is needed to achieve the required threshold processing of SUB1 targets.
Author response: The presented data does not suggest that the exoneme discharge events several hours prior to egress are 'rare'. In fact, our data suggests that exoneme discharge occurs at a regular clip over these 3 hours. Newly included data suggest that these are not spurious discharge events (Fig. 5D, E). Using the weak base, ammonium chloride, suggests that mature exonemes are present several hours prior to egress (Fig. 5D, E). These studies suggest the possibility that exocytosis and PVM rounding as well as rupture may not be directly coupled as previously assumed.

3. Lines 181-82. Ref. 12 is a review and might not be the most appropriate for statements about

the spatio-temporal relationship between exoneme exocytosis and merozoite egress. PMID: 28683142 uses transfectant clones with conditional KO of SERA genes, Western blotting for activation of SUB1 proteolytic activity against its targets, kinetics of egress in living parasites after washout of C1 and C2 to implicate rapid egress (< 8 min) upon washout of PKG inhibitors. Thus, it seems a bit unfair to say that the kinetics of exoneme exocytosis and subsequent egress have only been studied with fixed parasites.
Author response: We have modified the statement. Western blotting is an averaging experiment, and these experiments established the proteolytic activity of SUB1 against its targets. The use of inhibitors masks a critical aspect of the process, exocytosis. It assumes that proteolytic activity and exocytosis are the identical and occur together, i.e., if SUB1/PMX are released in the PV, they are active. However, our data suggests a different model, where exocytosis and proteolytic activity are decoupled. While the use of PKG inhibitors provide critical insights, they are also misleading in that they suggest that nothing happens prior to 8 minutes before egress. Further experimentation without the use of inhibitors, perhaps using a fluorescent reporter assay for PMX/SUB1 activity in the PV may help resolve these differences and will be the focus of our future studies.

4. Lines 150-151: "These data suggest that exoneme exocytosis occurs all over the schizont and may not be limited to a single exit site or specific cellular location within the merozoite." As there are multiple merozoites within a schizont and there is significant Brownian/active movement of merozoites in live recordings, this statement should be removed or significantly toned down.
Author response: Done.

5. Please revise Lines 392-393. Ref. 31 applied BaF for only 2 h and did not examine egress vs. invasion specifically as they examined only the result with Giemsa staining after 6 h.
Author response: Done.

Also, please correct this citation from BioRxiv to JBC as this paper has been published for some time - PMID: 39084459
Author response: We apologise for this mistake. The citation has been corrected.

Reviewer #2: 1. Line EXP2mRuby3/PMXSEP is clonal and seems to grow normally but a growth curve should be shown (supplementary data).
Author response: Done (Suppl. Fig. 1).

Also was ATc added after transfection for a brief time or was it present throughout culturing?
Author response: ATC was added to the media and used throughout culturing.

The authors briefly mention presence of ATc but this should be explained in more detail as one would assume that the presence or absence of ATc even briefly might affect PMX levels?
Author response: We added text to clarify that parasites were growing in aTc media throughout culturing. The reviewer is correct. If parasites were grown in the absence of atc, the PMX expression is suppressed.

2. Genotyping PCRs showing absence of the endogenous locus in line EXP2mRuby3/PMXSEP should be provided as supplementary material.
Author response: We prefer to have these genotyping PCRs in the main figure.

3. Line 304: "To test this hypothesis, we first generated PfERC conditional mutants using the glucosamine (GlcN) responsive ribozyme system (Fig. 6A)". The authors have already generated that line for their previous publication focusing on PfERC. Is this a new one? Is the genetic background different? Please clarify.
Author response: We have generated PfERC conditional mutants in the NF54 *attb* background. They differ from the previous publication, in which PfERC conditional mutants were modified in the 3D7 background.

4. Line 322-323: "The addition of GlcN to PfERC-glmS/PMXSEP and PfERC-M9/PMXSEP parasites also resulted in loss of PMX processing (Fig. 6B)." One would expect that addition of GlcN to PfERC-M9/PMXSEP would have no effect on PMX processing as PfERC levels remain unaffected. Can the authors clarify this?
Author response: The statement has been modified. The reviewer is correct, the cleavage activity of the glmS ribozyme depends on GlcN. The M9 is a mutated glmS ribozyme incapable of cleaving and serves as a robust control when using GlcN in the ribozyme knockdown system.

5. Figure 7, lines 460-471: Although I agree with the concept of two independent signals for egress, irrelevant of the timing, there are data that suggest that exoneme exocytosis might not be Calcium dependent (Balestra et al, 2021) and Calcium flux is correlated only with microneme release. One would think that if there are 2 calcium fluxes even from distinct stores they would have probably been observed in studies with Fluo4 or Calcium reporters that have been used in Plasmodium, unless they occur at the same time. Can the authors elaborate on their rational for this model?
Author response: This is a very interesting question raised by the reviewer. There are a few possibilities. Prior studies using calcium reporters or Fluo4 have only looked at time points closer to egress (10-15 min before egress) and utilized PKG inhibitors. Another issue here would be

that Fluo4 as well as calcium reporters may not be sensitive enough to pick up every calcium spike and may themselves interfere with calcium in the cytoplasm. Clearly, our data suggest further investigation is needed to define the role of calcium signals in the asexual life cycle of Plasmodium parasites. Furthermore, the study by Balestra showed that knockout of ICM1 did not inhibit SUB1 exocytosis but this also assumes ICM1 is the only pathway for calcium release from stores in parasites. It is possible that other pathways for calcium mobilization exist in the parasite. The rationale behind our model is that exocytosis is clearly PKG dependent and so far, the preponderance of literature suggests that cGMP signals and calcium signals are co-dependent. We agree with the reviewer that our prior assumptions may be incorrect and therefore, we have modified the model slightly to leave a question around calcium at this earlier timepoint as our data does not assess calcium mobilization at this time.

Reviewer #3: Fig 2 A - please mention total number of schizonts from which percentages were calculated
Author response: The total number of schizonts was added as suggested.

Fig 5 should also have the C1 panel for comparison.
Author response: We have added a panel with the PKG inhibitor to Figure 5 as the reviewer suggested.

Fig 6C and movies 8 and 9. - Has the fluorescence channel been merged with DIC? could the background speckles be reduced for better clarity?
Author response: The fluorescence channel has not been merged with the DIC. We have reduced the background as suggested by the reviewer.

"The data shows" and "These data show" are a bit overused throughout the manuscript. Perhaps omit saying this.
Author response: The statement has been modified.

A lot of statements are repeated in the Introduction, Results and Discussion. Perhaps reducing this would make the text more crisp and clear.
Author response: Edited for clarity.

Line 70-79 seems out of place here. Could be omitted in the Intro and appear only when relevant in the Results when talking about ERC.
Author response: Edited for clarity.

Typos - "Intergrants" in Fig 1B

Author response: We apologize for this mistake. The text has been corrected.

---

1. Lopes da Silva M, Thieleke-Matos C, Cabrita-Santos L, Ramalho JS, Wavre-Shapton ST, Futter CE, et al. The host endocytic pathway is essential for Plasmodium berghei late liver stage development: Plasmodium interaction with host endocytic pathway. Traffic. 2012;13: 1351–1363.

2. McCloy RA, Rogers S, Caldon CE, Lorca T, Castro A, Burgess A. Partial inhibition of Cdk1 in G 2 phase overrides the SAC and decouples mitotic events. Cell Cycle. 2014;13: 1400–1412.

3. Jakic B, Buszko M, Cappellano G, Wick G. Elevated sodium leads to the increased expression of HSP60 and induces apoptosis in HUVECs. PLoS One. 2017;12: e0179383.

4. Ressurreição M, Thomas JA, Nofal SD, Flueck C, Moon RW, Baker DA, et al. Use of a highly specific kinase inhibitor for rapid, simple and precise synchronization of Plasmodium falciparum and Plasmodium knowlesi asexual blood-stage parasites. PLoS One. 2020;15: e0235798.

5. Baker DA, Stewart LB, Large JM, Bowyer PW, Ansell KH, Jiménez-Díaz MB, et al. A potent series targeting the malarial cGMP-dependent protein kinase clears infection and blocks transmission. Nat Commun. 2017;8: 430.

6. Vanaerschot M, Murithi JM, Pasaje CFA, Ghidelli-Disse S, Dwomoh L, Bird M, et al. Inhibition of resistance-refractory P. falciparum kinase PKG delivers prophylactic, blood stage, and transmission-blocking antiplasmodial activity. Cell Chem Biol. 2020;27: 806–816.e8.

7. Prommana P, Uthaipibull C, Wongsombat C, Kamchonwongpaisan S, Yuthavong Y, Knuepfer E, et al. Inducible knockdown of Plasmodium gene expression using the glmS ribozyme. PLoS One. 2013;8: e73783.

8. Fierro MA, Asady B, Brooks CF, Cobb DW, Villegas A, Moreno SNJ, et al. An Endoplasmic Reticulum CREC Family Protein Regulates the Egress Proteolytic Cascade in Malaria Parasites. MBio. 2020;11. doi:10.1128/mBio.03078-19

9. Kudyba HM, Cobb DW, Vega-Rodríguez J, Muralidharan V. Some conditions apply: Systems for studying Plasmodium falciparum protein function. PLoS Pathog. 2021;17: e1009442.

10. Reeder JC, Cowman AF, Davern KM, Beeson JG, Thompson JK, Rogerson SJ, et al. The

adhesion of Plasmodium falciparum-infected erythrocytes to chondroitin sulfate A is mediated by P. falciparum erythrocyte membrane protein 1. Proc Natl Acad Sci U S A. 1999;96: 5198–5202.

11. Salanti A, Staalsoe T, Lavstsen T, Jensen ATR, Sowa MPK, Arnot DE, et al. Selective upregulation of a single distinctly structured var gene in chondroitin sulphate A-adhering Plasmodium falciparum involved in pregnancy-associated malaria. Mol Microbiol. 2003;49: 179–191.

12. Küster N, Roling L, Ouayoue A, Steeg K, Przyborski JM. A systematic targeted genetic screen identifies proteins involved in cytoadherence of the malaria parasite P. falciparum. Mol Microbiol. 2025;123: 330–343.

13. Nasamu AS, Glushakova S, Russo I, Vaupel B, Oksman A, Kim AS, et al. Plasmepsins IX and X are essential and druggable mediators of malaria parasite egress and invasion. Science. 2017;358: 518–522.

14. Triglia T, Scally S, Seager B, Pasternak M, Dagley L, Cowman A. Plasmepsin X activates function of the PCRCR complex in P. falciparum by processing PfRh5 for binding to basigin and invasion of human erythrocytes. Research Square. 2023. doi:10.21203/rs.3.rs-2410384/v1

15. Mukherjee S, Nguyen S, Sharma E, Goldberg DE. Maturation and substrate processing topography of the Plasmodium falciparum invasion/egress protease plasmepsin X. Nat Commun. 2022;13: 4537.

16. Martinez M, Bouillon A, Brûlé S, Raynal B, Haouz A, Alzari PM, et al. Prodomain-driven enzyme dimerization: a pH-dependent autoinhibition mechanism that controls Plasmodium Sub1 activity before merozoite egress. MBio. 2024; e0019824.

17. Glushakova S, Beck JR, Garten M, Busse BL, Nasamu AS, Tenkova-Heuser T, et al. Rounding precedes rupture and breakdown of vacuolar membranes minutes before malaria parasite egress from erythrocytes. Cell Microbiol. 2018;20: e12868.

18. Tan MSY, Koussis K, Withers-Martinez C, Howell SA, Thomas JA, Hackett F, et al. Autocatalytic activation of a malarial egress protease is druggable and requires a protein cofactor. EMBO J. 2021;40: e107226.

19. Geoghegan ND, Evelyn C, Whitehead LW, Pasternak M, McDonald P, Triglia T, et al. 4D analysis of malaria parasite invasion offers insights into erythrocyte membrane remodeling and parasitophorous vacuole formation. Nat Commun. 2021;12: 1–16.
