# Supplementary material for: Spatiotemporal dynamics of signal dependent exocytosis and parasitophorous vacuolar membrane rupture during Plasmodium falciparum egress
Source: PLoS Pathog. 2026 May 11;22(5):e1014214. doi: 10.1371/journal.ppat.1014214 (PMC13183284; doi:10.1371/journal.ppat.1014214)
Supplement: Attachment — Submitted filename: ReviewerResponse2.docx [file ppat.1014214.s022.docx]

**Additional Editor Comments:**

Your manuscript has been re-evaluated by the same three reviewers, who acknowledge that it has substantially improved. Nevertheless, reviewers 1 and 2 remain unconvinced that the PMX-SEP fluorescent signal observed upon packaging into exonemes cannot originate from the cytosol, nor that secretion is definitively demonstrated. They therefore request additional experimental evidence to directly address these points. While such experiments may present technical challenges, they would be essential to rule out alternative interpretations of the data and to robustly support the proposed model. Accordingly, we recommend strengthening the manuscript by providing additional experimental validation of the model presented in Figure 1A.

Response: We thank you for the opportunity to revise the manuscript. From the comments provided to us, it appears that reviewers 2 and 3 have questions regarding the secretion vs acidification of exonemes. Reviewers 1 and 3 agree with the major conclusions in our prior revision. This resubmission includes new data (Fig. 6 and Movie S8-9) showing that exonemes are acidified several hours prior to exocytosis. Furthermore, the fluorescence signals prior to vesicle acidification are insensitive to PKG inhibition, unlike exocytosis. These data directly address the key points raised by reviewers 2 and 3 regarding the relationship between PKG inhibition and acidification. We hope that this revision will be acceptable for publication.

**Reviewers' Comments:**

Reviewer's Responses to Questions

**Part I - Summary**
Please use this section to discuss strengths/weaknesses of study, novelty/significance, general execution and scholarship.

Reviewer #1: The authors have done an excellent job addressing all raised concerns. The addition of NH4Cl treatment to identify acidified exonemes prior to discharge is an elegant control. The conclusions have been toned down appropriately. The manuscript will add significantly to our understanding of merozoite maturation, PVM rupture, and egress, events that are important targets for therapy development.

Response: We thank the reviewer for their positive assessment of our resubmission.

Reviewer #2: Dedkhad et al, provided an updated version of the manuscript which includes some additional data regarding the timing of exoneme exocytosis. However, the authors did not address many of my concerns experimentally and although the technical challenges are substantial the data presented only partially support the claims made. With the use of inhibitors (which probably do not reflect what happens in real time) this is a process that can be completed in 15 minutes, but the authors suggest that the parasite under normal conditions does it in 3h. Although I find the model very exciting clearly pointing to diverse signalling events to regulate every process, I respectfully disagree with the authors that detailed characterisation on what the fluorescent signals are should be the focus of future studies.

Response: We thank the reviewer for their assessment. We have included new data that show that vesicle acidification occurs several hours prior to the initial exocytosis events (Fig. 6 and Movie S8-9). Importantly, these acidification events are insensitive to PKG inhibition unlike exocytosis. We have also tried to ensure that our conclusions are consistent with the data as well as prior literature.

Reviewer #3: I thank the authors for considering the issues raised and addressing them with more data and clarifications.

Response: We thank the reviewer for their careful consideration of our resubmission.

**Part II – Major Issues: Key Experiments Required for Acceptance**
Please use this section to detail the key new experiments or modifications of existing experiments that should be absolutely required to validate study conclusions.

Generally, there should be no more than 3 such required experiments or major modifications for a "Major Revision" recommendation. If more than 3 experiments are necessary to validate the study conclusions, then you are encouraged to recommend "Reject".

Reviewer #1: (No Response)

Reviewer #2: My main concern still remains that the authors cannot provide data to distinguish if the fluorescent puncta is PMX secreted in the PV or PMX in non-acidified exonemes/micronemes. Can the authors exclude the possibility that this acidic organelles via a PKG regulated mechanism change their pH? The ammonium chloride experiments show that these compartments become fluorescent (i.e with neutral pH). Does this suggest exoneme secretion? If not, the parasite could trigger de-acidification in a PKG dependent manner to stop PMX activity as prolonged protease activity could lead to non-specific cleavage.

Response: We respectfully disagree with the hypothesis that the fluorescent puncta were due to neutralization of vesicles and that this was caused by PKG inhibition. This is because such a model is contradicted by a large body of data in prior literature (and reproduced by several groups around the world) showing that PKG inhibition blocks exocytosis. Again, the suggestion that ammonium chloride causes exoneme secretion also contradicts an extensive body of published data in both mammalian cells as well as parasites showing NH_4_Cl acts as a weak base to neutralize pH in intracellular compartments.

We have included new data that show that acidification of exonemes occurs several hours prior to exocytosis (Fig 6 and Movie S8-9). The data further show that exoneme acidification is insensitive to PKG inhibition (Fig. 6A-C), demonstrating the PKG does not regulate a change in pH. The most parsimonious explanation of these data is that PKG does not trigger de-acidification. The ammonium chloride experiments only suggest that vesicle pH neutralization alters PMX^SEP^ fluorescence since ammonium chloride is a weak base. If it triggers exoneme exocytosis, then this would suggest the presence of a very novel ammonium chloride sensing signaling pathway that overcomes PKG inhibition. There is no other evidence in literature for such a pathway, and previous studies have used ammonium chloride to neutralize acidic compartments in *P. falciparum* without triggering egress (Kuhn et al Cell. Microbiol. 2007, 9:1004-1013; Walsh et al PLoS Path. (2025) 21(7): e1012779).

Regarding the localisation of PMX, Mukherjee at al (Nat. Commun. 2022) analysed in detail via EM colocalisation of PMX and SUB1 with AMA1 and rhopty markers, however they only speculated about a "precursor compartment" as no data were provided for that. Distribution of these proteins and potential overlap in these organelles is still under investigation.

Response: We agree that the data presented in Mukherjee et al is consistent with a precursor compartment and further studies are needed to clarify the changing compartments during parasite development.

The authors state that they do not have any PMX or SUB1, nor MSP1 or SERA5 antibodies, which I agree makes it very difficult to study some of the events in detail, although they have used these antibodies in the past. However, they do have a PMX-V5 tagged line where they could have looked for PV localised PMX in a time course experiment either by WB or IFA.

Response: We used these antibodies in the past resulting in the complete depletion of our antibody stocks. As I’m sure the reviewer is aware, certain labs in the field have closed (due to retirements) and due to issues with shipping antibodies into the US, we have been unable to source more of these antibodies.

There are two major issues that prevent us from directly testing the dual localization of PMX at this time. 1) The vast majority of PMX is within exonemes and as our data show, there is a constant and slow release of PMX over the final hours of schizogony (Fig.1R A, B). Only a small proportion of the total cellular PMX is in the PV at this time. Thus, the signal from PMX in exonemes is much stronger than any signal from the PV. If we increase signal, then we observe so much non-specific signal that we can conclude PMX localizes everywhere within the infected RBC. We show data here from early and late schizonts (Fig.1R). These IFA data show that some PMX may co-localize with the plasma membrane marker, MSP1. However, the resolution of IFA is not sufficient to make that conclusion especially since we observe plenty of signal within the parasite as well. While these data can be used to argue for the dual localization, we do not think that it conclusively proves that PMX localizes to the PV in early schizonts.

2) Further, as these experiments average over the total population, the signal on immunoblots are also swamped by schizonts that haven’t yet embarked on exocytosis. Another possibility (though unlikely) is that upon exocytosis, the epitope tag may also be cleaved leading to loss of signal. At this time, we do not have a way to assess the loss of tag. Published data also suggest that proteases may form macromolecular aggregates in the PV (Tan et al EMBO J, 2021, 40: e107226), which may make their isolation and localization difficult via western blots.

Based on prior published data as well as our data that PKG inhibition blocks exocytosis and our data the PKG inhibition does not block vesicle acidification, the simplest model that is consistent our data is that exocytosis occurs in ~44 h.p.i schizonts.

A better way to test this observation in the future would be to target a split-GFP system to the PV but our prior attempts at using the split-GFP system in *P. falciparum* did not result in any fluorescence. We are now attempting to use a split-turboID system to send one half of TurboID to the PV and the other half will be fused to PMX. Thus, if the two halves of TurboID are only reconstituted in the PV (and not during transit in the secretory pathway), then we will be able to observe biotinylation of proteins in the PV. This enzymatic approach will hopefully prove successful. However, assuming that the split-turboID system works as designed, it will take us at least a year to optimize this approach and will be beyond the scope of this manuscript. The data in this manuscript is consistent with secretion ~3 hours prior to the egress and our measured conclusions reflect that.

Figure 1R. Representative immunofluorescence assay (IFA) images of PfERC-HA/PMX V5 late (A, 48 h.p.i) and mid (B, 44 h.p.i) schizont in 3D7 parental line stained with anti-merozoite surface protein 1 (MSP1, magenta), anti-V5 (PMX, cyan), DAPI (DNA, yellow), and Phase-contrast (DIC). Scale bar, 5 μm.

Reviewer #3: I appreciate the limitations posed by non-availability of valid exonemal markers to validate their system and to confirm correct subcellular localisation of PMX-SEP. However, I still think the system would greatly benefit from direct validation of the model in Figure 1A, to confirm that the fluorescence we see in the videos are triggered by a change in pH specifically upon exoneme exocytosis. While the authors say the PMX-SEP is non-fluorescent until exocytosis, and that a punctate pattern is observed within the enclosed PVM, none of the videos rule out cytosolic origin of these signals when PMX is packaged into exonemes nor do they effectively show localisation to the PPM. So, either a non-pH sensing PMX-tag control (in which we would see a contrasting, more stable signal throughout compared to PMX-SEP, and possibly reacts differently to C1 treatment and ERC knockdown) or any other form of similar control experiment would help prove these are authentic exocytosis events, clarify certain questions about the timing of exocytosis and would further bolster the paper in my opinion. On the other hand, since treatments with C1, ammonium chloride and ERC knockdown do have an effect on PMX-SEP that supports their model, it is reasonable to say that the evidence supports the conclusions as of now.
Response: We thank the reviewer for recognizing the limitation in the system and agree that more markers are needed for exonemes. We are also happy to note that the reviewer agrees that the data supports our conclusions.

The new data showing that exoneme acidification occurs several hours prior to exocytosis bolsters our conclusions (Fig. 6 and Movie S8-9). Importantly, the acidification of vesicles is insensitive to PKG inhibition, unlike the exocytosis events which are blocked by PKG inhibition (Fig. 6A-C). Prior studies have shown that PKG regulates exocytosis and our data show that PKG inhibition blocks PMX^SEP^ fluorescence observed in later schizonts. Furthermore, we observed that vesicle acidification occurs at ~40 h.p.i (PKG insensitive) and we did not observe any fluorescence again until ~44 h.p.i (PKG sensitive) (Figs. 5 and 6). Neutralizing the pH using ammonium chloride results in consistent stable signal regardless of PKG inhibition, further supporting the conclusion that the events observed are the result of exocytosis.

Together these data strongly suggest that the events we are observing are exocytosis and not simply de-acidification or PMX^SEP^ being packaged into new vesicles (which we now show is insensitive to PKG inhibition These data (Fig. 6) as well as prior literature are consistent with the model that the fluorescence observed is due to vesicle exocytosis and not simply neutralization of the pH via unknown mechanisms leading to cytosolic origin of these signals.

To clarify my point about E64 - in videos such as Movie 3, we cease to see PMX-SEP and EXP2-mRuby signals upon egress because the cell has burst and merozoites dispersed. E64 might allow us more time to observe these signals after exoneme release has been triggered. Do merozoites continue exoneme release after practically been "released" from the PV? However, I agree with the authors that it might not add significant more detail.

The authors have satisfactorily resolved other issues by adding more videos and schizont numbers to support their conclusions.

Response: We are thankful that the reviewer agrees that the data support our conclusions.

**Part III – Minor Issues: Editorial and Data Presentation Modifications**
Please use this section for editorial suggestions as well as relatively minor modifications of existing data that would enhance clarity.

Reviewer #1: (No Response)

Reviewer #2: 1. Regarding the timing of exocytosis, the authors state that “Another possibility is that the
observed PMXSEP fluorescence at this earlier timepoint could be secretory vesicles that were
not yet acidified and therefore fluorescent.” (lines 262-263).
Author response: Newly included data using ammonium chloride is inconsistent with this
hypothesis. Therefore, we have removed this line from the manuscript.
This has not been removed from the revised manuscript (lines 259-260)

Response: We have included this sentence in this revision as a way to transition to our data showing that vesicle acidification occurs earlier in schizogony and is not blocked by PKG inhibition.

2. Figure legend for 2E and 2F is missing

Response: We apologize for this mistake. The figure legends are now included.

3. For the ammonium chloride experiments, the authors reference Lopes da Silva M et al. in their rebuttal letter. In that study Ammonium chloride was used for disruption of host liver cell acidification, which is not mentioned. Furthermore I could not find that reference in the revised manuscript, yet the authors reference Shadija N et al, and I could not find in which experiment they used Ammonium chloride.

Response: We were referring to the Lopes da Silva et al since they used NH_4_Cl in the context of *Plasmodium* parasites. As the reviewer identifies, they used it in host liver cell acidification, hence we removed it in the revision. We apologize for the miscommunication, Shadija et al was miscited, they used Bafilomycin in their experiments. Ammonium chloride has been utilized across many systems including *Plasmodium* to neutralize intracellular acidic compartments (Kuhn et al Cell. Microbiol. 2007, 9:1004-1013; Walsh et al PLoS Path. (2025) 21(7):
e1012779).

4.Line 296 "Calcium signals are known to be essential for exoneme exocytosis". That is not the case. "They might be important" is more accurate.

Response: We have modified this sentence. It now says ‘Calcium signals have been suggested to be essential for exoneme exocytosis..”

5. Since the proteases essential for egress are activated by low pH[15,35–38] (lines 386-387). SUB1 is active at neutral pH

Response: Based on prior data (Mukherjee et al., mBio, (2023)[;](https://doi.org/10.1128/mbio.00673-23) Martinez et al., mBio, (2024); 15:e00198-24).SUB1 is inhibited by its propiece (p31). The SUB1 propiece is cleaved by PMX, which has optimal activity at low pH. Thus, while SUB1 is active at neutral pH, it is activated only upon PMX cleavage of the pro-piece at low pH.

Reviewer #3: (No Response)
